# Potential link between Fusobacterium enrichment and DNA methylation accumulation in the inflammatory colonic mucosa in ulcerative colitis

**DOI:** 10.18632/oncotarget.18716

**Published:** 2017-06-27

**Authors:** Tomomitsu Tahara, Ichiro Hirata, Naoko Nakano, Sayumi Tahara, Noriyuki Horiguchi, Tomohiko Kawamura, Masaaki Okubo, Takamitsu Ishizuka, Hyuga Yamada, Dai Yoshida, Takafumi Ohmori, Kohei Maeda, Naruomi Komura, Hirokazu Ikuno, Yasutaka Jodai, Toshiaki Kamano, Mitsuo Nagasaka, Yoshihito Nakagawa, Tetsuya Tuskamoto, Makoto Urano, Tomoyuki Shibata, Makoto Kuroda, Naoki Ohmiya

**Affiliations:** ^1^ Department of Gastroenterology, Fujita Health University School of Medicine, Toyoake, Japan; ^2^ Department of Gastroenterology, Kenporen Osaka Central Hospital Japan, Osaka, Japan; ^3^ Department of Diagnostic Pathology I, School of Medicine, Fujita Health University, Toyoake, Japan

**Keywords:** DNA methylation, colonic mucosa, ulcerative colitis, *Fusobacterium*, genome-wide methylation

## Abstract

**BACKGROUND AND AIM:**

*Fusobacterium* enrichment has been associated with colorectal cancer development. Ulcerative colitis (UC) associated tumorigenesis is characterized as high degree of methylation accumulation through continuous colonic inflammation. The aim of this study was to investigate a potential link between *Fusobacterium* enrichment and DNA methylation accumulation in the inflammatory colonic mucosa in UC.

**METHODS:**

In the candidate analysis, inflamed colonic mucosa from 86 UC patients were characterized the methylation status of colorectal a panel of cancer related 24 genes. In the genome-wide analysis, an Infinium HumanMethylation450 BeadChip array was utilized to characterize the methylation status of >450,000 CpG sites for fourteen UC patients. Results were correlated with *Fusobacterium* status.

**RESULTS:**

UC with *Fusobacterium* enrichment (FB-high) was characterized as high degree of type C (for cancer-specific) methylation compared to other (FB-low/neg) samples (*P*<0.01). Genes hypermethylated in FB-high samples included well-known type C genes in colorectal cancer, such as *MINT2* and *31*, *P16* and *NEUROG1*. Multivariate analysis demonstrated that the FB high status held an increased likelihood for methylation high as an independent factor (odds ratio: 16.18, 95% confidence interval: 1.94-135.2, *P*=0.01). Genome-wide methylation analysis demonstrated a unique methylome signature of FB-high cases irrespective of promoter, outside promoter, CpG and non-CpG sites. Group of promoter CpG sites that were exclusively hypermethylated in FB-high cases significantly codified the genes related to the catalytic activity (*P*=0.039).

**CONCLUSION:**

Our findings suggest that *Fusobacterium* accelerates DNA methylation in specific groups of genes in the inflammatory colonic mucosa in UC.

## INTRODUCTION

The non-spore-forming, anaerobic Gram-negative bacteria, *Fusobacterium* is part of the normal flora in the human mouth and gut mucosa. Although *Fusobacterium* species are part of the gut microbiome in human, their invasive [1,2], adherent [3,4], and pro-inflammatory [5–7] features have been noted. *Fusobacterium* have been associated with inflammatory disorders such as periodontitis [8], cerebral abscesses [9], acute appendicitis [10] and inflammatory bowel diseases [1,11,12]. Moreover, emerging evidence suggest a possible link between *Fusobacterium* infection and colorectal carcinogenesis through altering the host immune responses [13–16]. Enrichment of *Fusobacterium* have been especially associated with colorectal cancers and adenomas with methylation phenotypes, suggesting the potential role of this bacteria in DNA methylation related colorectal tumorigenesis [17, 18].

Since *Fusobacterium* has a reported association with inflammatory bowel diseases (IBD), including both ulcerative colitis (UC) and Crohn’s diseases [1, 11, 12], and IBD is one of the highest risk factors for colorectal cancer [19]. In particular, UC associated colorectal cancers are characterized as high degree of methylation accumulation through continuous colonic inflammation [20, 21]. The aim of this study was to investigate a potential link between *Fusobacterium* enrichment and DNA methylation accumulation in the inflammatory colonic mucosa in UC.

## RESULTS

### Detection of fusobacterium in the inflammatory colonic mucosa in UC patients and its association with methylation status of candidate genes

**Figure 1:**
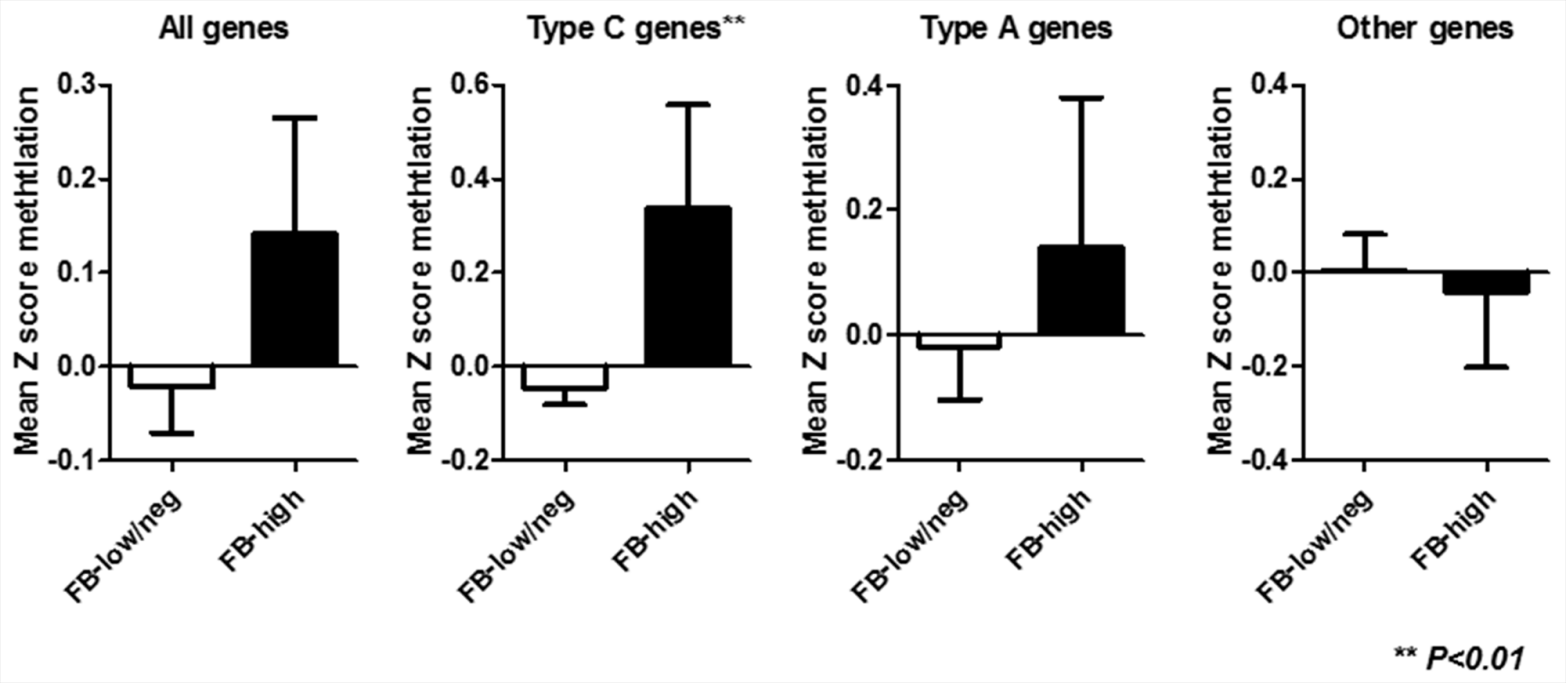
Mean Z score methylation of all, type C, type A and other genes in relation to the *Fusobacterium* status. FB-low/neg*, Fusobacterium* low and negative samples; FB-high, *Fusobacterium* high samples; Statistical analysis was performed using Student’s t-test

**Figure 2:**
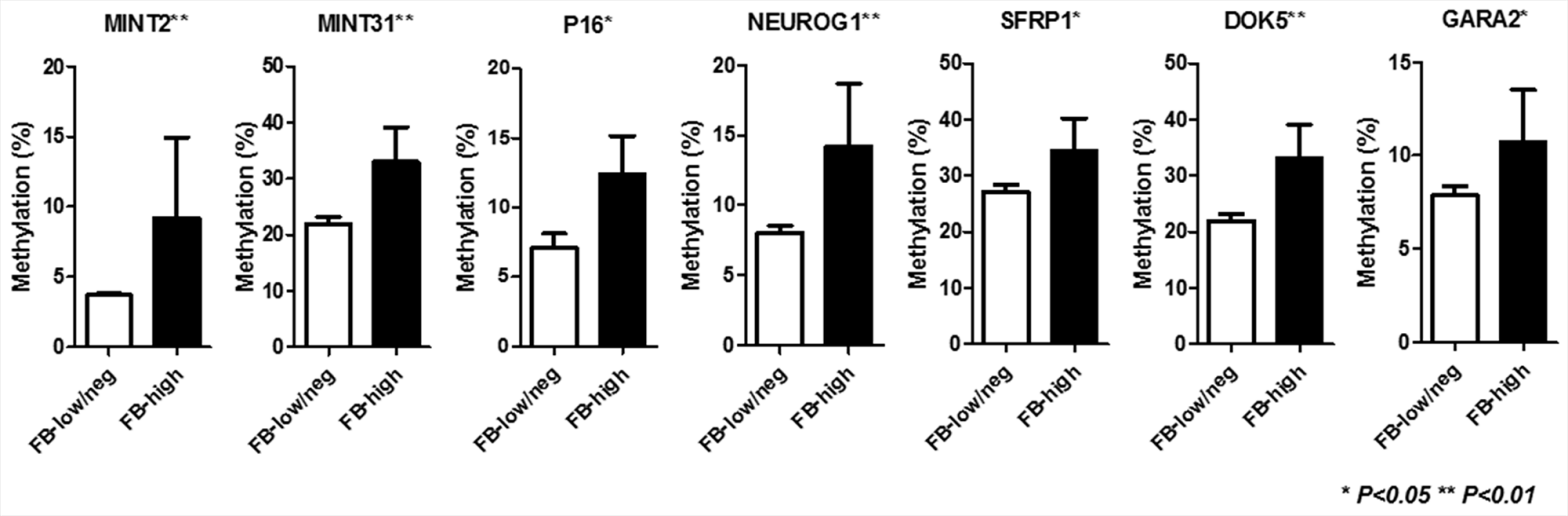
Methylation status of specific genes in relation to the *Fusobacterium status.* FB-low/neg*, Fusobacterium* low and negative samples; FB-high, *Fusobacterium* high samples; The statistical analysis was performed using Student’s t-test

Among 86 inflammatory colonic mucosa from UC patients, pan-fusobacterium was heavily enriched in ten (11.6%) cases using the same cut off value of our recent study [22]. Supplementary Figure 1 illustrates the results of an unsupervised clustering analysis based on the methylation status of a panel of 24 candidate genes. This analysis showed that samples with *Fusobacterium* high (FB-high) distributed as moderately methylated samples but were not clustered clearly each other. Since the analyzed genes can be divided into type A (for age-related: *N33*, *MYOD1*, *ER1*, *HPP1*, and *SFRP1)*, type C (for cancer-specific: *MINT1, 2, 12, and 31, RASSF1A*, *P16*, *NEOUROG1*, *TERT*, and *MGMT*) and other colorectal cancer related (*GARA2*, *IGF2*, *DPYS*, *NKX2-5*, *DOK5*, *RARB2*, *SLC16A12*, *CDH13* and *SPOCK2*) genes [23–26]. We then divided analyzed genes into these groups in relation to the *Fusobacterium* status. This analysis demonstrated that the mean methylation Z score of type C genes was significantly higher among FB-high group compared to *Fusobacterium* low and negative (FB-low/neg) group (*P*<0.01). On the other hand, no significant associations between mean methylation Z score of all, type A and other genes and *Fusobacterium* status were observed (Figure 1). Analysis of individual panel showed significantly higher methylation of known type C genes in colorectal cancer such as (*MINT2* and *31*, *P16* and *NEUROG1*) in FB high samples [23, 24], while subset of type A and other colorectal cancer related genes (*SFRP1*, *DOK5*, *GARA2*) [24, 25, 26] also presented significantly higher methylation in the FB-high samples (Figure 2). On the other hand, we did not find genes hypermethylated in FB-low/neg samples among these candidate panels (data not shown). We also evaluated the methylation status of the *LINE1* repetitive element, which is an indicator for genome wide hypomethylation [27]. However, we did not observe significant association between *LINE1* methylation status and *Fusobacterium* status (Supplementary Figure 2).

Since methylation status of colonic mucosa in UC patients would be influenced by the clinicopathological factors [28]. We then performed multivariate analysis assessing the factors related to the hypermethylation of type C genes in the colonic mucosa in UC patients. Age, gender, duration, extension of disease, clinical course, number of hospitalization, presence of refractory or steroid dependency were included for this analysis with the *Fusobacterium* status. The mean Z score methylation of type C genes in the colonic mucosa in UC patients presented an approximately Gaussian distribution, with over representation of hypermethylated cases, we set cut-off value of 0.18 (mean Z score methylation) for the definition samples with hypermethylated (methylation high) cases. The result demonstrated that FB-high status held an increased likelihood for methylation high as an independent factor (odds ratio: 16.18, 95% confidence interval: 1.94-135.2, *P*=0.01), while other clinicopethological factors were not significantly associated with methylation high cases (Table 1).

**Table 1:** Multivariate analysis assessing the factors related to the methylation-high* in type C genes

Variables	Odds ratio (95% confidence interval)	P value
Age (40y =<)	1.68 (0.39-7.17)	0.49
Gender (female)	5.39 (0.94-30.91)	0.06
Duration (10y=<)	0.58 (0.10-3.23)	0.53
Extension (total colitis)	1.49 (0.31-7.18)	0.62
Clinical course (flare-up)	0.42 (0.04-4.36)	0.47
Number of hospitalization (2=<)	0.91 (0.16-5.14)	0.91
Refractory	1.35 (0.17-10.59)	0.78
Steroid dependency	1.84 (0.16-21.05)	0.62
FB-high	16.18 (1.94-135.2)	0.01

^*^Methylation-high, mean Z score of methylation>0.18;

### The genome-wide methylation status of CpG islands distinguishes FB-high cases

**Figure 3:**
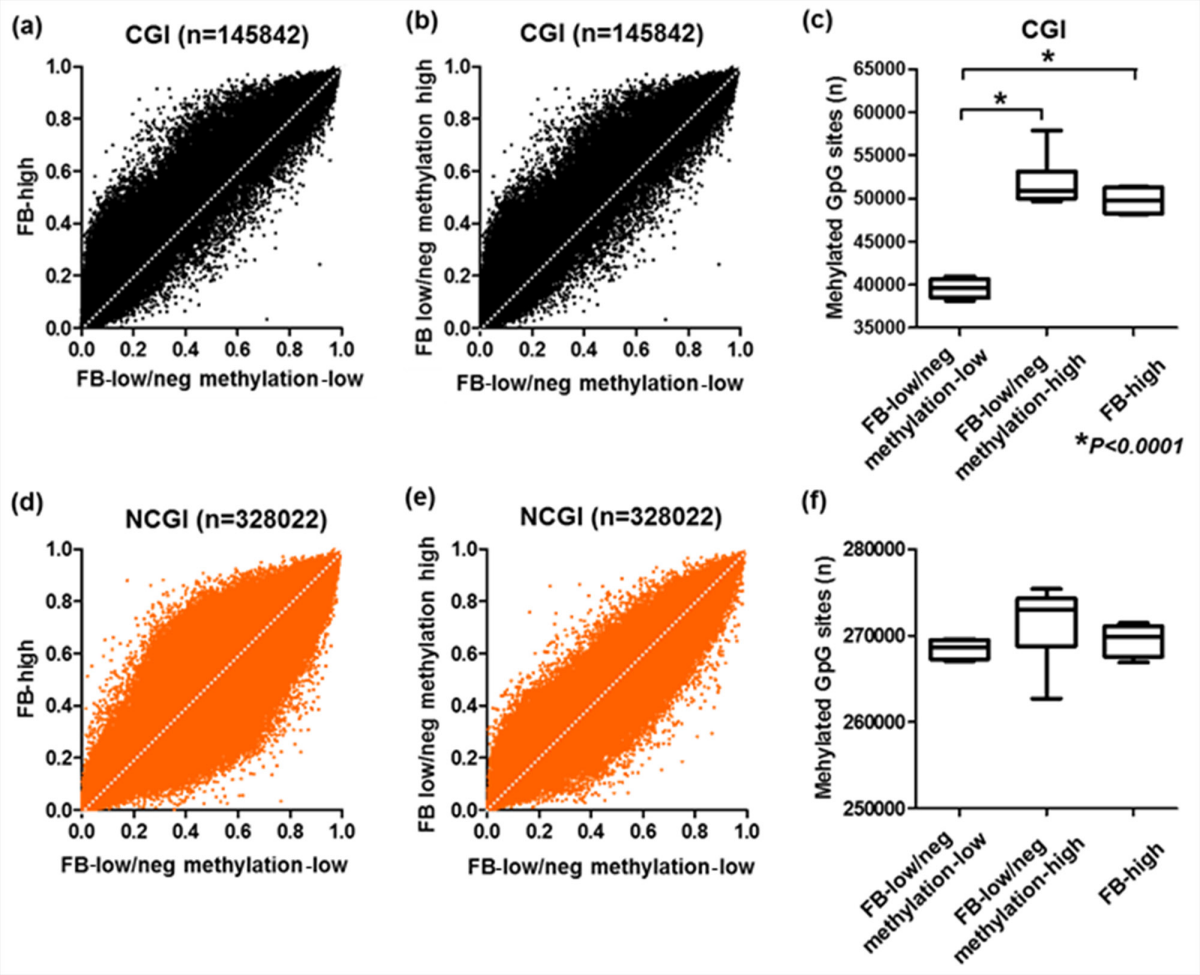
Genome scale analysis using HumanMethylation450 BeadChip array Comparison of mean methylationβ-value of *Fusobacterium* high (FB-high) samples, *Fusobacterium* low and negative (FB-low/neg) with methylation low samples and *Fusobacterium* low and negative (FB-low/neg) with methylation-high samples **(A)-(D)**. Mean number of methylated sites among FB-high, FB-low/neg methylation-low and FB-low/neg methylation-high samples **(E) (F)**. All sites were divided into CpG islands (CGI, upper) and non-CpG islands (NCGI, lower). The statistical analysis was performed Student’s t-Test.

To determine the methylation changes occurred in the colonic inflammatory mucosa of the UC patients with FB-high cases, we used an Infinium HumanMethylation450 BeadChip array, which allowed us to query methylation status of >450,000 CpG sites within the genome. The Infinium HumanMethylation450 BeadChip array data were available for ten patients [28], all these were considered to be FB-low/neg cases. The methylation levels of FB low/neg cases were considerably different among the samples (Supplementary Figure 1). Among the ten samples of these the Infinium HumanMethylation450 BeadChip array data were available, 6 and 4 belonged to hypermethylated (methylation-high) and hypomethylated cluster (methylation-low) based on the unsupervised clustering analysis of 24 candidate panels (Suppelementary Figure 1). We then included additional four genomic samples from FB-high cases for the analysis. Based on GRCh37/hg19, we first checked the methylation status of 473,864 CpG sites and divided the sites into CpG islands (CGI: n=145,842) and non-CpG islands (NCGI: n=328,022). We found that accelerated methylation among the FB-low/neg methylation-high samples compared to the FB-low/neg methylation-low samples especially at the CGI sites rather than the NCGI sites. Similar result was also observed for the comparison of FB-high samples and FB-low/neg methylation-low samples (Figure 3). When a gain in methylation was defined as a methylation level ≥20% (β-value≥0.2), the numbers of methylated sites in the CGI was significantly greater in both FB-low/neg methylation-high and FB-high samples compared to the FB-low/neg methylation-low samples (Both *P*<0.0001), while the numbers of methylated sites was not significantly different among the FB-low/neg methylation-high and FB-high samples (*P*>0.1) (Figure 3). On the other hand, we did not observe any significant association between the numbers of methylated sites among three groups in the NCGI (all *P*>0.1) (Figure 3).

**Figure 4:**
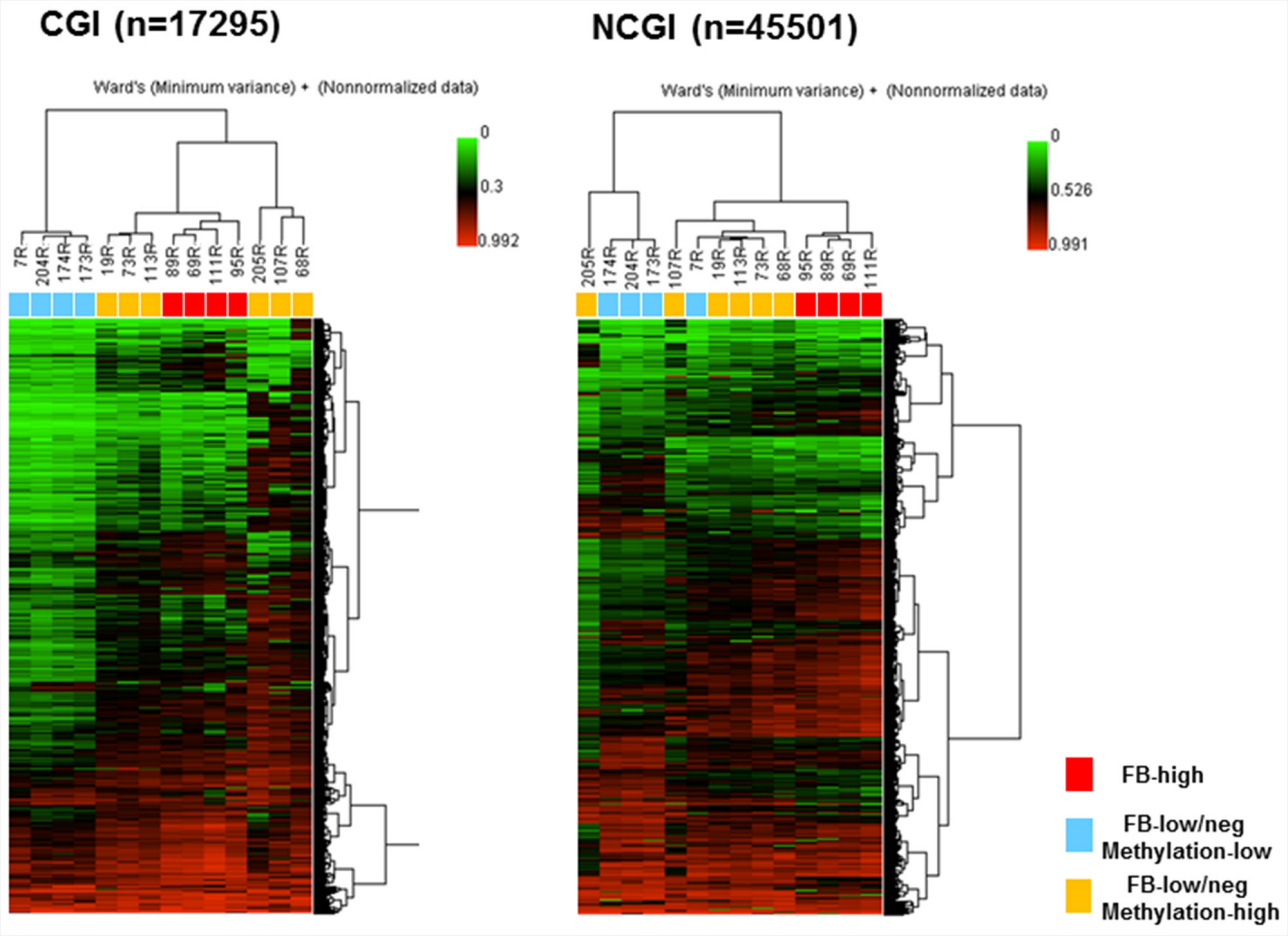
Unsupervised hierarchical clustering analysis of CpG islands (CGI, left) and non-CpG islands (NCGI, right) using 10% most variant probes among fourteen UC patients Red boxes, *Fusobacterium* high (FB-high) samples; Blue boxes, *Fusobacterium* low and negative (FB-low/neg) with methylation low samples; Yellow boxes, *Fusobacterium* low and negative (FB-low/neg) with methylation high samples; Samples ID number were listed above the boxes.

We next aimed to investigate whether the methylated sites in FB-high group would be exclusive in this group compared to the FB-low/neg groups. Unsupervised clustering analysis using the 10% of the probes with the greatest variation very clearly distinguished the samples from the FB-high cases in both the CGI and NCGI. FB-high cases were clustered together as the moderately methylated samples in the CGI and also clustered together as the hypermethylated samples in the NCGI (Figure 4). The tight cluster of the FB-high cases as the moderately methylated samples was also confirmed when dividing the CGI into the promoter CGI (PCGI: n=1684) and outside the promoter CGI (NPCGI: n=15611) (Supplementary Figure 3). On the other hand, dividing NCGI into the promoter NCGI (PNCGI: n=3231) and outside the promoter NCGI (NPNCGI: n=42270) showed that FB high cases were clustered as hypomethylated samples in the PNCGI, while the same samples were clustered as hypermethylated samples in the NPNCGI (Supplementary Figure 3).

### Identification of the hypermethylated promoter CpG islands in the severe phenotypes

**Figure 5:**
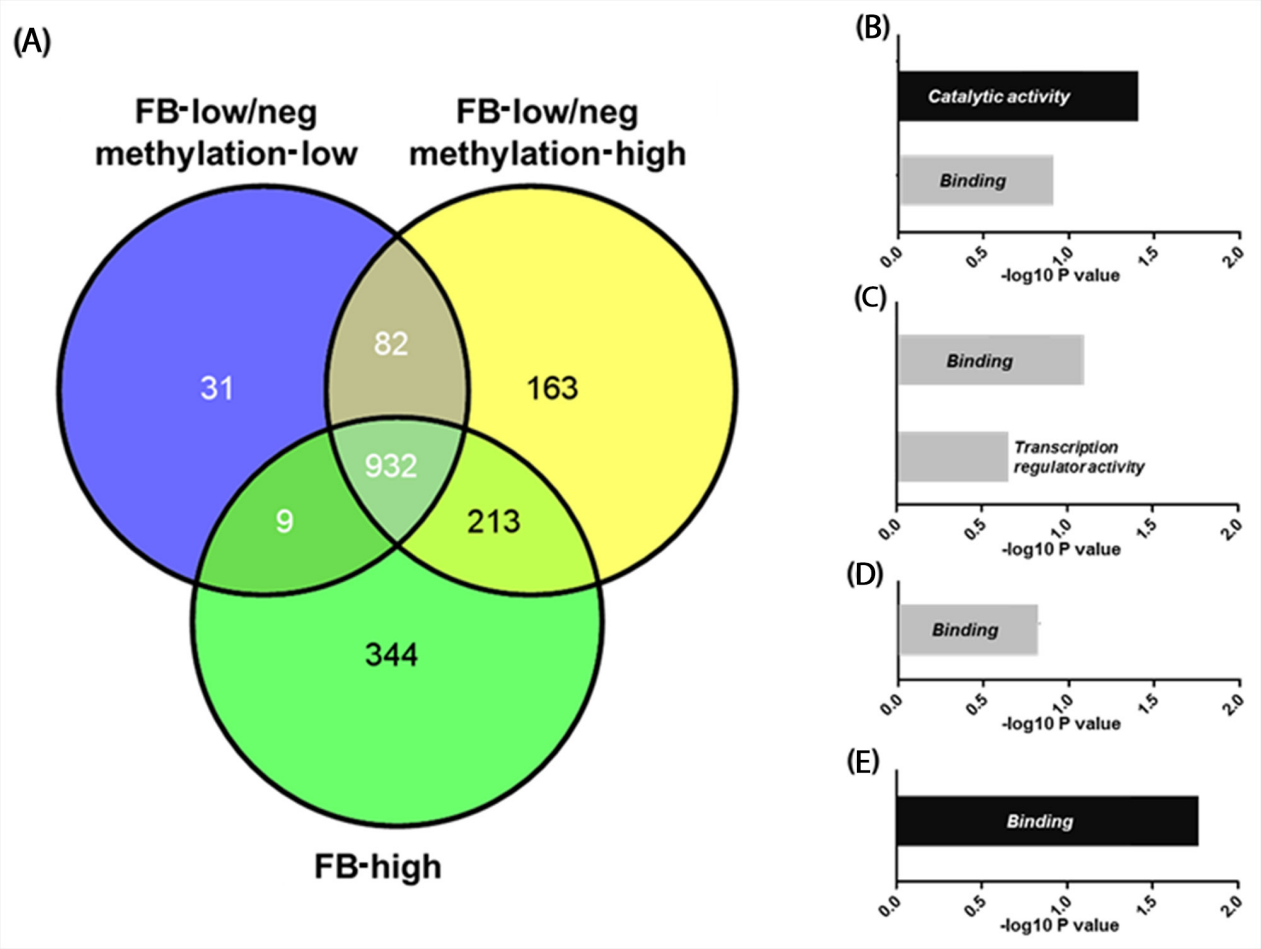
Venn-diagram representing the number of genes methylated in *Fusobacterium* high (FB-high), *Fusobacterium* low and negative (FB-low/neg) with methylation low and *Fusobacterium* low and negative (FB-low/neg) with methylation high samples **(A).** Results of the gene ontology analysis of the genes methylated exclusively in the specific groups or common elements **(B)-(E)**. Genes methylated exclusively in FB-high groups (B), common elements in FB-low/neg methylation high and FB-high groups (C), common elements in FB-low/neg methylation low and FB-low/neg methylation high groups (D), and common elements in all three groups (E). Categories with significant enrichment by the Benjamini method are shown by black boxes, while no significant enrichment are shown by grey boxes.

We next explored genes that were exclusively hypermethylated in FB-high cases. For this analysis, we focused on the PCGIs because of their influence on gene expression. When a gain in methylation was defined as a methylation level ≥20% (β-value≥0.2), we identified 344 genes that were exclusively hypermethylated in the FB-high group. Gene ontology analysis using DAVID revealed that these genes are significantly codified genes related to the catalytic activity (*P*=0.039: Figure 5 and Supplementary Table 2 and 3). Gene ontology analysis using methylated genes in the common elements in all three groups (FB-high, FB-low/neg methylation-low and high groups) showed that these genes are significantly codified genes related to the binding (*P*=0.016: Figure 5 and Supplementary Tables 2 and 3). On the other hand, methylated genes among other groups or elements did not show any significant functional enrichment using the same analysis.

## DISCUSSION

An unsupervised clustering analysis based on the methylation status of a panel of 24 candidate genes showed that FB-high cases were distributed as moderately methylated samples but were not clustered clearly. However, the FB-high cases presented hypermethylation especially in the type C genes, including *MINT2* and *31*, *P16* and *NEUROG1*, these methylation have been reported in the colorectal cancers with methylation phenotype [23, 24]. Multivariate analysis with adjustment of clinicopathological factors demonstrated that FB-high held an increased likelihood for hypermethylation of the type C genes.

Enrichment of *Fusobacterium* has been reported in the colorectal cancer and adenoma tissues especially in cases with methylation phenotype [17, 18]. The methylation phenotype of colorectal cancers are characterized as accumulation of cancer specific methylation of type C genes [23, 24]. The current potential association between *Fusobacterium* enrichment and methylation of type C genes in inflamed mucosa of UC might provide evidence, supporting the potential link between *Fusobacterium* and methylation related carcinogenesis in UC. *Fusobacterium* have been associated with several inflammatory diseases such as such as periodontitis [8], cerebral abscesses [9], acute appendicitis [10] and inflammatory bowel diseases [1, 11, 12]. It is interesting to note that the colorectal cancer with methylation phenotype that is associated with *Fusobacterium* have a distinct immune response with abundant tumor infiltrating lymphocytes. This inflammatory reaction has been thought to be a host immune response to the tumor cells [29, 30]. It has been also shown that the infection of *Fusobacterium* accelerates the pro-inflammatory cytokine expressions in adenoma tissues in the mice model [16]. Since chronic inflammation induced by pro-inflammatory cytokines have roles in the methylation induction [21]. It is reasonable to expect that *Fusobacterium* alters inflammatory immune response and influence the methylation status in UC patients.

On the other hand, UC associated colorectal cancer and its background mucosa have been characterized as hypermethylation in type A genes [20]. It has also been reported that methylation phenotypes are rather rare in UC associated colorectal cancers [31–32]. Whether hypermethylation of type C genes in the inflamed mucosa in UC linked to the future risk of colorectal cancer is currently unknown. The role of *Fusobacterium* enrichment in UC associated colorectal cancer is not clearly demonstrated, while our previous study demonstrated that Japanese UC patients with *Fusobacterium* enrichment was associated with chronic continuous inflammation in the colonic mucosa [22], which may link to accelerated DNA methylation and colorectal cancer. Potential role hypermethylation of type C genes in UC with FB high cases need to be further evaluated in relation to their biological and clinical significances.

A unique methylome signature of UC with FB-high cases were also highlighted through the genome-wide methylation analysis. Compared to the FB-low/neg methylation-low cases, methylation accumulation shown in FB-high cases seemed to be more striking at CGI, rather than the NCGI, which appeared to be similar to that of FB-low/neg methylation high samples. On the other hand, FB-high cases were very tightly clustered as unique samples including PCGI, NPCGI, PNCGI and NPCGI by the clustering analysis of the 10% of the probes with the greatest variation. We also showed that group of PCGIs that were exclusively hypermethylated in FB-high cases significantly codified the genes related to the catalytic activity. The enrichment of genes related to the catalytic activity was not observed in other groups of samples nor their common elements, suggesting that *Fusobacterium* accelerates DNA methylation in specific groups of genes. Although the association between genes related to the catalytic activity and UC associated carcinogenesis remain unknown, methylated genes in FB-high cases included several cancer related genes, for example, *DAG1* and *RBM7.* Deletion of *DAG1* is associated to the worse outcome of breast cancer patients [33]. Lacking of *RBM7* has been also reported to be associated with DNA damage hypersensitivity that can linked to cancer predisposition [34]. Enrichment of *Fusobacterium* has been associated with colorectal and pancreatic cancers [13, 14, 35]. The discovery of the specific methylation induction in *Fusobacterium* emphasizes the importance of an improved understanding of pathway-specific molecular changes in UC associated carcinogenesis and raises the possibility that specific epigenetic therapies that target alterations in proteins related to catalytic activity could be useful in the treatment and chemoprevention of UC associated colorectal cancer. In the same time, our findings also deserve to be tested in animal models, where one could specifically explore the possibility of therapeutic intervention modulating DNA methylation with antibiotics, anti-inflammatory or demethylation agents in the prevention or treatment of UC associated colorectal cancer.

## MATERIALS AND METHODS

### Tissue samples

We used genomic DNA samples of 86 UC patients who underwent colonoscopy at the Fujita Health University Hospital (Toyoake, Japan). All of the samples were extracted from fresh frozen endoscopic biopsies taken from inflamed mucosae of the rectum. These patients included 48 males and 38 females. The median age and clinical duration were 35 and 4.5 years, respectively. Regarding their clinical course, 9 patients presented only one attack and the remaining cases showed at least one time flare-up of disease. Five and two cases eventually underwent surgery due to toxic megacolon and UC-associated colorectal cancer, respectively. All cases were clinically in remission at the time of endoscopy. The histopathological examinations revealed mild or moderate inflammation but no evidence of dysplasia or neoplasia at any of the sites from which the biopsies were taken. Based on the appearances during endoscopy, 19 patients exhibited inflammatory mucosae only in the rectum, and 25 patients exhibited extensions of the inflammatory mucosae into the left side of the colon (sigmoid and descending colons). The remaining 41 patients exhibited extensions of the inflammatory mucosae into proximal sites (the transverse and ascending colons and the cecum). This cohort was recruited from our previous studies that investigated the associations of DNA methylation with clinical phenotypes, host genetic factors and telomere lengths [28, 36–38]. This study was approved by the Human Research Ethics Committee of the Fujita Health University School of Medicine. Each participant provided written informed consent for the use of his or her clinical and laboratory data for publication and research purposes. The study was conducted according to the principles outlined in the Declaration of Helsinki.

### Quantitative PCR analysis for fusobacterium

Quantitative real-time PCR analysis for Fusobacterium was performed using the Universal PCR Master Mix (Bio-Rad) and StepOnePlus™ Real-Time PCR System (Applied Biosystems). The pan-fusobacterium TaqMan primer/probe set used in this study were described previously [13, 39]. The cycle threshold (Ct) values for pan-fusobacterium were normalized to the amount of human DNA in each reaction by using a primer/probe set for the reference gene, prostaglandin transporter (PGT), as described previously [40]. All assays were done in duplicate and we averaged the results. We have reported that subset of UC cases show heavy enrichment of *Fusobacterium* in the inflamed colonic mucosa [22]. In this cohort, we identified ten cases (11.6%) of UC with enrichment of *Fusobacterium* using the same cut off value [22]. We then defined these cases as *Fusobacterium* high (FB-high) cases. Since the amount of *Fusobacterium* in detectable cases except the FB-high cases was much lower than that of FB-high cases and had no relevance to the clinic-pathological features of patients [22], we then attached these cases with *Fusobacterium* undetectable cases and defined as *Fusobacterium* low and negative (FB-low/neg) cases.

### CpG methylation analysis of candidate panels for colorectal cancer

For this cohort, we have characterized the methylation status of candidate 45 CpG islands, in relation to their clinicopethological features [28]. Among the 45 genes, we selected a panel of 24 genes that were reported to be associated with colorectal carcinogenesis [23–26]. In colorectal cancer, there appears to be two types of methylation that are associated with cancer progression: type A (for age-related) methylation, and type C (for cancer-specific) methylation [41]. Based on this, we selected 9 and 5 genes that are reported to be associated with type A and type C methylation, respectively [23–26]. The type A genes included *N33*, *MYOD1*, *ER1*, *HPP1*, and *SFRP1* [23, 24]. The type C genes included *MINT1, 2, 12, and 31, RASSF1A*, *P16*, *NEOUROG1*, *TERT*, and *MGMT* [23, 24]. We also included other 9 genes (*GARA2*, *IGF2*, *DPYS*, *NKX2-5*, *DOK5*, *RARB2*, *SLC16A12*, *CDH13* and *SPOCK2*) that have been associated with colorectal tumorigenesis in human or mice [25, 26]. We also evaluated the methylation status of the *LINE1* repetitive element. All the methylation analysis was performed by the bisulfite pyrosequencing. The bisulfite treatment of the DNA was performed with an EpiTect bisulfite kit (Qiagen, Tokyo, Japan) according to the manufacturer’s protocol. Pyrosequencing was performed using a PSQ24 system with a Pyro-Gold reagent kit (QIAGEN, Tokyo, Japan), and the results were analyzed using PyroMark Q24 software (QIAGEN). List of the genes and the primers used for pyrosequencing are listed in Supplementary Table 1.

### Genome-wide methylation analysis

We performed array-based DNA methylation analyses using the Infinium HumanMethylation450 BeadChip array, which allowed us to query the methylation status of >450,000 CpG sites within the genome and to cover 99% of the RefSeq genes. Genomic DNA samples from the inflamed rectal mucosae of fourteen UC patients were used for this analysis. Among them, the data were available for ten patients [28]. All these ten cases were considered to be *Fusobacterium* low and negative (FB-low/neg) case. We then used additional four genomic samples from *Fusobacterium* high (FB-high) cases for the Infinium HumanMethylation450 BeadChip array experiment. Bisulfite modification of the genomic DNA was performed using an EZ DNA Methylation Kit (Zymo Research). The bisulfite conversion efficiency was determined based on sample-dependent controls on the chip and was displayed in the quality control panel in the software. All samples passed the quality control measurements. The samples were run on an Infinium HumanMethylation450K BeadChip (Illumina) and scanned on an Illumina iScan instrument according to the manufacturer’s instructions. The methylation values for the individual CpG sites in each sample were obtained as β-values. The β-value generated for each CpG locus reflected a measure of the intensities of the methylated (β = 1) and unmethylated probes (β = 0). The β-values are continuous variable that are calculated by dividing the intensity of the methylated beads by the combined intensity, and the resultant values range from 0 to 1. The genomic regions were defined according to National Center for Biotechnology Information coordinates, which were downloaded from the University of California, Santa Cruz website in February 2009 (GRCh37/hg19). We removed probes that were targeted for an annotated SNP (dbSNP137) and for either the X or Y chromosome. Information about the CpG islands and promoters (surrounding gene transcription start sites) was also obtained based on the GRCh37/hg19.

### Clustering analysis

Unsupervised clustering analysis using ArrayTrack™ (http://www.fda.gov/ScienceResearch/BioinformaticsTools/Arraytrack/) was performed to identify distinct subgroups based on the methylation status.

### Gene ontology analysis

Functional enrichment of the methylated genes was determined by gene ontology analysis using DAVID Bioinformatics Resources 6.7 (http://david.abcc.ncifcrf.gov/). P-values were corrected for multiple hypotheses testing using the Benjamini method.

### Statistical analysis

The continuous variables were compared between two and more groups using Student’s t-test. P values <0.05 were considered statistically significant.

## SUPPLEMENTARY MATERIALS FIGURES AND TABLES









## Abbreviations

UC: Ulcerative colitis
IBD: inflammatory bowel diseases
FB-high: *Fusobacterium* high
FB-low/neg: *Fusobacterium* low and negative
