# Supplementary material for: Potential link between Fusobacterium enrichment and DNA methylation accumulation in the inflammatory colonic mucosa in ulcerative colitis
Source: Oncotarget. 2017 Jun 27;8(37):61917–26. doi: 10.18632/oncotarget.18716 (PMC5617474; doi:10.18632/oncotarget.18716)
Supplement: Supplementary file 1 [file oncotarget-08-61917-s001.pdf]

# Potential link between Fusobacterium enrichment and DNA methylation accumulation in the inflammatory colonic mucosa in ulcerative colitis

## SUPPLEMENTARY MATERIALS

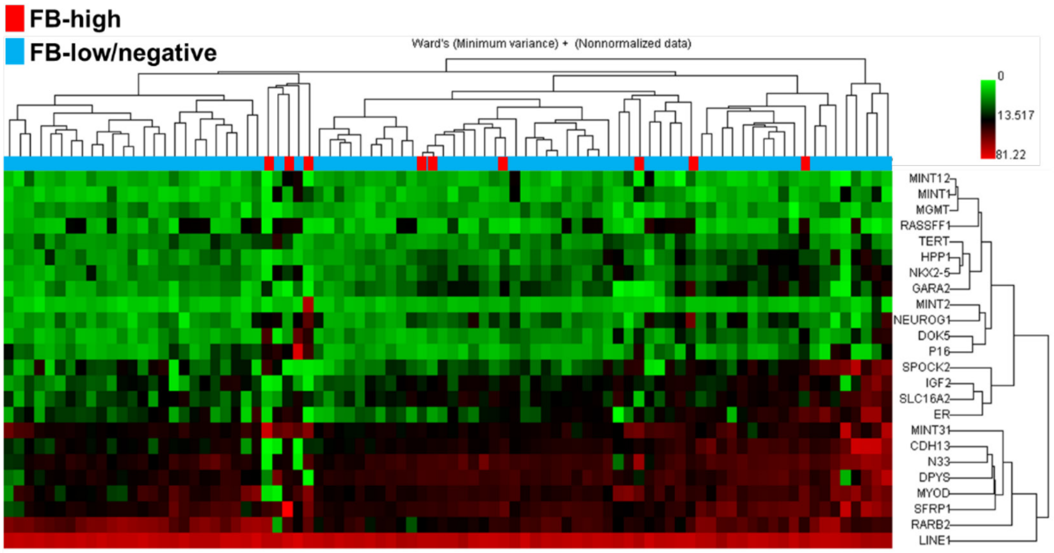


**Supplementary Figure 1: Unsupervised hierarchical clustering analysis of 24 candidate panels using inflamed colonic mucosa from ten UC patients.** Blue boxes, *Fusobacterium* low and negative (FB-low/neg) samples; Red boxes, *Fusobacterium* high (FB-high) samples;

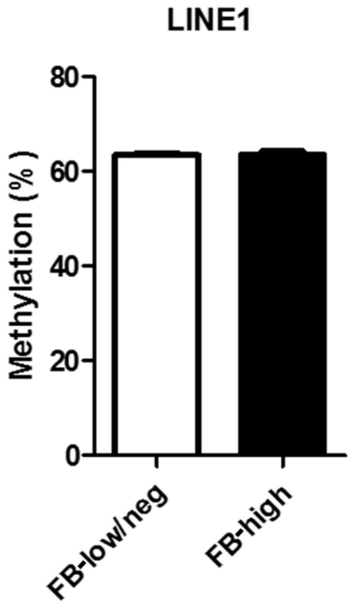


**Supplementary Figure 2: Methylation status of *LINE1* repetitive element among *Fusobacterium* low and negative (FB-low/neg) and *Fusobacterium* high (FB-high) samples.** The statistical analysis was performed using Student's t-test.

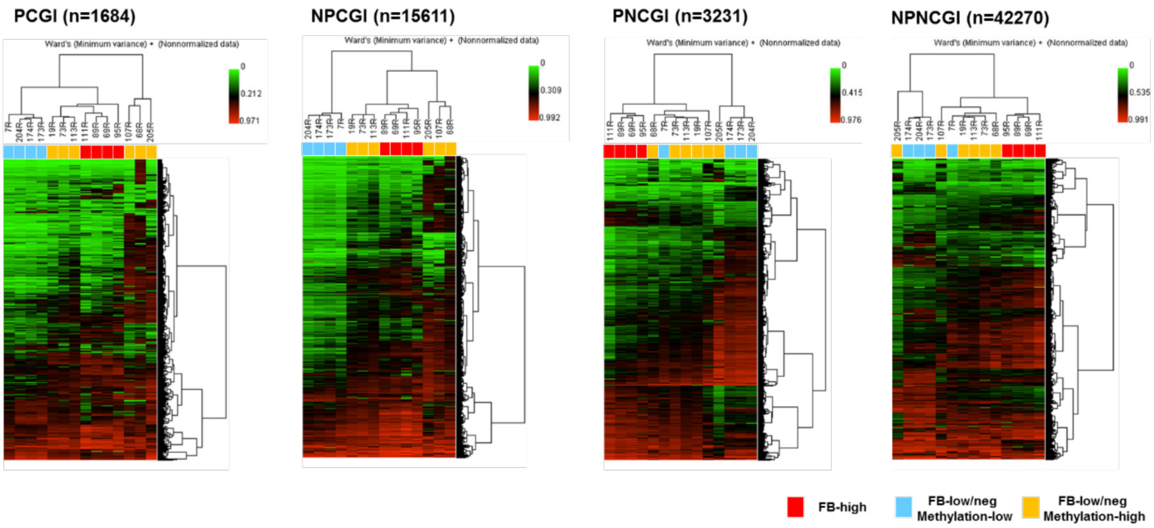


**Supplementary Figure 3: Unsupervised hierarchical clustering analysis using 10% most variant probes among fourteen UC patients.** PCGI, promoter CpG sites; NPCGI, outside promoter CpG sites; PNCGI, promoter non-CpG sites; NPNCGI, outside promoter non-CpG sites; Red boxes, *Fusobacterium* high (FB-high) samples; Blue boxes, *Fusobacterium* low and negative (FB-low/neg) with methylation-low samples; Yellow boxes, *Fusobacterium* low and negative (FB-low/neg) with methylation-high samples; Samples ID number were listed above the boxes.

**Supplementary Table 1: List of candidate genes analyzed in this study and primer sequences used in pyrosequencing.**

**See Supplementary File1**

**Supplementary Table 2: Comparison of methylated genes in FB-high, FB-low/neg methylation-low and FB-ow/neg methylation-high groups.**

**See Supplementary File2**

**Supplementary Table 3: Summary of gene ontology analysis using methylated genes in specific groups**

**See Supplementary File3**
